# Assessment of the Particularities of Thrombophilia in the Management of Pregnant Women in the Western Part of Romania

**DOI:** 10.3390/medicina59050851

**Published:** 2023-04-28

**Authors:** Miruna Samfireag, Cristina Potre, Ovidiu Potre, Lavinia-Cristina Moleriu, Izabella Petre, Ema Borsi, Teodora Hoinoiu, Marius Preda, Tudor-Alexandru Popoiu, Andrei Anghel

**Affiliations:** 1 Department of Internal Medicine, Discipline of Clinical Practical Skills, “Victor Babes” University of Medicine and Pharmacy, No. 2 Eftimie Murgu Square, 300041 Timisoara, Romania; samfireag.miruna@umft.ro (M.S.); tstoichitoiu@umft.ro (T.H.); 2 Advanced Cardiology and Hemostaseology Research Center, “Victor Babes” University of Medicine and Pharmacy, No. 2 Eftimie Murgu Square, 300041 Timisoara, Romania; 3 Department of Internal Medicine, Discipline of Hematology, “Victor Babes” University of Medicine and Pharmacy, No. 2 Eftimie Murgu Square, 300041 Timisoara, Romania; potre.ovidiu@umft.ro (O.P.); borsi.ema@umft.ro (E.B.); 4 Department III of Functional Sciences, Discipline of Medical Informatics and Biostatistics, “Victor Babes” University of Medicine and Pharmacy, No. 2 Eftimie Murgu Square, 300041 Timisoara, Romania; moleriu.lavinia@umft.ro (L.-C.M.); tudor.popoiu@student.umft.ro (T.-A.P.); 5 Department XII of Obstetrics and Gynaecology, Discipline III of Obstetrics and Gynaecology, “Victor Babes” University of Medicine and Pharmacy, No. 2 Eftimie Murgu Square, 300041 Timisoara, Romania; petre.izabella@umft.ro; 6 Department IX of Surgery I, Discipline II of Surgical Semiology, “Victor Babes” University of Medicine and Pharmacy, No. 2 Eftimie Murgu Square, 300041 Timisoara, Romania; marius.preda@umft.ro; 7 Department of Biochemistry and Pharmacology, Discipline of Biochemistry, “Victor Babes” University of Medicine and Pharmacy, No. 2 Eftimie Murgu Square, 300041 Timisoara, Romania; biochim@umft.ro

**Keywords:** thrombophilia, pregnant women, miscarriage

## Abstract

*Background and objectives*: Thrombophilia in pregnant women is a condition whose incidence is constantly increasing worldwide, and, under these conditions, the development of preventive procedures is becoming essential. In this study, we aimed to evaluate thrombophilia in pregnant women in the western part of Romania and to establish anthropometric characteristics, socioeconomic features, and genetic and risk factors. *Material and Methods*: 178 pregnant women were divided into three study groups, according to the type of thrombophilia, aiming to carry out the genetic profile and the acquired one. Anthropometric measures and biological tests were performed. *Results*: The mixed type of thrombophilia predominates. The particularities of pregnant women diagnosed with thrombophilia are higher age, living in an urban environment, with normal BMI, approximately 36 weeks of gestational period, and having at least one miscarriage. Regarding the most frequent thrombophilic genetic markers, we obtained the MTFHR gene mutation C677T and A1298C, followed by the PAI-1 4G/5G gene mutation. Smoking represents an aggravating factor in the evolution of this pathology, manifested through the increase of D-dimers and the decrease in antithrombin values, simultaneously with the increase in therapeutic need. *Conclusions*: The predominance of MTHFR and PAI-1 4G/5G gene polymorphism is a particularity of pregnant women with thrombophilia from the western part of Romania. Smoking is confirmed as an important risk factor in spontaneous abortion.

## 1. Introduction

Thrombophilias can be categorized as either acquired or inherited [1]. Mixed thrombophilia is a condition that can result from both hereditary and nongenetic sources. The risk of venous thromboembolism (VTE) is associated with thrombophilia—a condition that ultimately results in thrombosis, particularly in women who are pregnant [2]. Although expensive, testing for thrombophilia entails a comprehensive range of coagulation and genetic tests, and interpretations call for clinical expertise [3]. Strong evidence linking unfavorable pregnancy outcomes with thrombophilia in pregnancy is limited. The most common factor in maternal thromboembolism is inherited thrombophilia. This is also linked to a higher risk of some unfavorable pregnancy outcomes, such as fetal loss in the second and third trimesters, abruptions, severe intrauterine growth restriction, and early-onset, severe preeclampsia [4,5]. The hemostatic system changes during pregnancy to become hypercoagulable, which raises the risk of thrombosis throughout pregnancy and reaches its peak at term [5]. One of the risk factors among reproductive diseases is represented by hereditary thrombophilia [1].

While acquired forms of thrombophilia are linked with both venous and arterial events, inherited forms are mostly linked with a tendency to VTE. Antiphospholipid antibodies (aPL) are what define antiphospholipid syndrome (APS) as an acquired form of thrombophilia, which is clinically indicated by arterial or venous thrombosis. The diagnosis is based on the Sydney criteria, which include one clinical criterion (pregnancy morbidity or serious or venous thrombosis) and one laboratory criterion (detection of abnormally high levels of high levels of IgM/IgG anticardiolipin antibodies, of anti-beta 2 glycoprotein-I antibodies, or of lupus anticoagulant). This is also linked to obstetric difficulties [1]. There are two categories of inheritable thrombophilic states, classified by Pinjala et al., namely, major or common inherited thrombophilias [6]. However, factor V Leiden and the prothrombin gene mutation G20210A are the most frequent causes of hereditary thrombophilia. Protein C and S and antithrombin abnormalities are less frequent but they represent the most severe triggers [6]. In addition, there is a connection between heritable thrombophilias and homozygosity for the methylene tetrahydrofolate reductase (MTHFR), which causes hyperhomocysteinemia, and poor pregnancy outcomes [5]. In addition to genetic thrombophilia, acquired hemostasis disorders can also result in hypercoagulable diseases. Due to increased procoagulant factors and decreased anticoagulants, as well as other alterations of the hemostasis, acquired states might maintain a prothrombotic situation [7]. Hyperhomocysteinemia, antiphospholipid antibody syndrome, increased levels of procoagulant factors, and decreased levels of anticoagulants are the main acquired diseases associated with thrombophilia [7]. A pregnant woman diagnosed with thrombophilia should be assessed for most risk factors, often referred to as triggers for first or recurrent thrombosis, while determining the best prophylaxis [7]. One identified pathogenic factor causing severe pregnancy problems is thrombophilia. Procoagulant factors that have undergone modifications—mutant genes with a high prevalence that raise the risk of developing thrombosis—have been researched [1,8,9]: MTHFR homozygous or heterozygous mutation in the C677T and A1298C positions, FVL gene homozygous or heterozygous mutation in the G1691A position, prothrombin gene homozygous or heterozygous mutation in the G20210A position, or the polymorphism of PAI-1 [1]. The hemostasis undergoes major alterations throughout a normal pregnancy, which favors procoagulants. On the other hand, during pregnancy, anticoagulant levels may slightly rise (in the case of tissue factor pathway inhibitor, or TFPI, the principal coagulation initiator), remain stable (in the case of antithrombin and protein C), or certainly decrease (protein S) [4]. A pregnancy’s favorable outcome is correlated with good placental development [10]. Women who have a prior history of pregnancy difficulties, notably recurrent loss or a prior stillbirth, are treated with a thromboprophylactic dose of low-molecular-weight heparin (LMWH). The use of aspirin and LMWH increases the chance of live births [5]; the follow-up is adjusted based on the side effects that may occur in each patient.

Either laboratory proof of a thrombophilic deficiency or discoveries of thrombotic alterations in placental histology specimens from the affected pregnancy serve as the foundation for this statement [5]. The use of medicine must begin early in pregnancy (preferably at six weeks gestation) before the main trophoblast invasion is complete to reap the full benefits of these treatment approaches [5]. Preterm birth, which affects 5–13% of deliveries in affluent nations, is a significant contributor to infant morbidity and mortality. Though the role of thrombophilia as a risk factor is unknown, genetic thrombophilia has the potential to induce preterm delivery [11]. Prematurity, whose causes are still unknown, is a public health issue due to its multifactorial nature, but also because of associated factors such as social class, demographics, biological, genetic, reproductive, environmental, behavioral, and psychosocial conditions. The accessibility to the quality of healthcare services [12] can also lead to premature birth. Newborns delivered before 37 weeks of pregnancy are considered preterm. Based on gestational age, preterm birth is divided into the following subcategories: extremely early (less than 28 weeks), very early (28 to 32 weeks), and moderate to late preterm (32 to 37 weeks) [13]. Premature newborns have more pronounced hemostatic system differences than term infants, although their hemostatic system development is hastened [14]. It is debatable as to how to treat expectant women who have thrombophilia. There is a wealth of research on the possible connection between thrombophilia and several obstetric problems. Placenta-mediated problems, miscarriages, and fetal losses are the most common obstetric complications that thrombophilia has been linked to [15].

This study aims to comprehensively evaluate thrombophilia in pregnant women in the western part of Romania, taking into account anthropometric characteristics—such as age, body mass index, gestational period, the weight of the newborns, socioeconomic, and risk factors and the genetic markers involved in thrombophilia.

## 2. Materials and Methods

We started our case-control study with 450 patients grouped into three samples: patients with inherited thrombophilia, acquired thrombophilia, and mixed (inherited and acquired) thrombophilia, with 150 individuals in each group. After applying the inclusion and exclusion criteria, we were left with 178 patients: based on the type of thrombophilia, we had 28.65% (51 patients) with inherited thrombophilia, 28.65% (51 patients) with acquired thrombophilia, and 42.7% (76 patients) with mixed (inherited and acquired) thrombophilia.

The thrombophilia-specific investigation panel aims to carry out the genetic profile and the acquired one: methylenetetrahydrofolate reductase (MTHFR) gene mutation, prothrombin (factor II) gene mutation, factor V Leiden gene mutation, plasminogen activator inhibitor gene mutation-1 (PAI-1), factor XIII gene mutation, glycoprotein IIb/IIIa gene mutation, fibrinogen gene mutation, the angiotensin converting enzyme gene mutation (ACE_insertion_deletion), the angiotensinogen gene mutation (AGT mutation M235T), the serine/threonine kinase gene mutation (ATR-1 Mutation A1166C), respectively, the Cystathionine Beta-Synthase gene mutation (CBS 844ins68), the evaluation of the status of antithrombin, lupus anticoagulant, anticardiolipin, and antiphospholipid antibodies, homocysteine, protein C, protein S, and of D dimers. Genetic testing for thrombophilia mutations entails the analysis of genes using various techniques such as DNA microarrays and real-time polymerase chain reactions.

The database was gathered using the Microsoft Excel software. For the statistical analysis we used two different software: JASPv16.4 and Microsoft Excel. After applying a descriptive analysis to our database, we applied the Shapiro–Wilk test to see the data distribution and decide upon the type of tests that would be used. The Mann–Whitney U-test was used to see if we had significant differences between the two different groups. The Kruskal–Wallis test was used when we analyzed medical tests between our three groups, and the Friedman test was applied to see the D-dimers’ value evolution during pregnancy. At the end of the study, we ran a regression analysis and calculated the correlation coefficients. The significance level was set at α=0.05 for the whole study. The noninvasive, case–control study was conducted on a cohort of 450 pregnant women, diagnosed with thrombophilia, in the western part of Romania, evaluated in routine clinical practice between 2018 and 2020. Because of the pandemic situation, in the last year of our study period (2020), the number of patients who sought proper care decreased significantly. The cohort was divided into three groups, depending on the type of thrombophilia. In the first group, 150 patients diagnosed with hereditary thrombophilia were enrolled. The second group of 150 patients was diagnosed with acquired thrombophilia. Finally, the third group consisted of 150 patients diagnosed with mixed thrombophilia. The study population included Caucasian women, with singleton pregnancy at the time of enrolment, with available results for inherited, acquired, and mixed thrombophilia, and with positive obstetrical history (recurrent pregnancy losses); the exclusion criteria were nonpregnant women, subjects with twin pregnancies, and pregnant women that had incomplete results for the thrombophilia screen. The use of the database was possible with the agreement of the Bioethics Commission of Victor Babes University of Medicine and Pharmacy, No. 2 Eftimie Murgu Square, Timisoara, Romania (51/28.09.2018); the informed consent was obtained from all subjects involved in the study. The study was conducted following the ethical principles set out in the Helsinki Declaration.

## 3. Results

**Figure 1:**
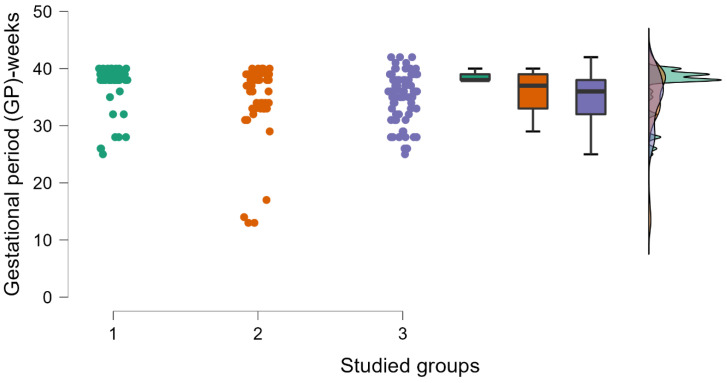
The gestational period is presented in three studied groups, using raincloud plots (in this type of graphical representation, we can see a complete data distribution, by density and by box plots). In group 1, the GP was significantly higher (*p* = 0.032 < 0.05, *p* value obtained from Kruskal–Wallis test; groups 1 and 2 contain 51 patients each, group 3 contains 76 patients).

**Figure 2:**
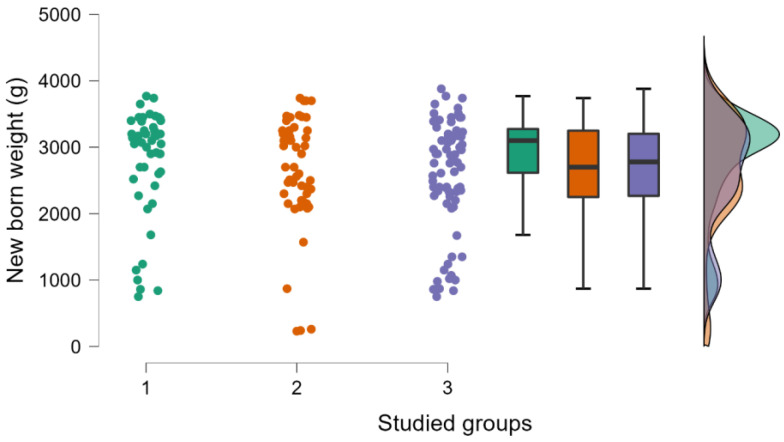
The newborn weight is presented in three studied groups, using raincloud plots; there were no statistically significant differences between the studied groups (*p* > 0.05, *p* value obtained from Kruskal–Wallis test; groups 1 and 2 contain 51 patients each, group 3 contains 76 patients).

**Figure 3:**
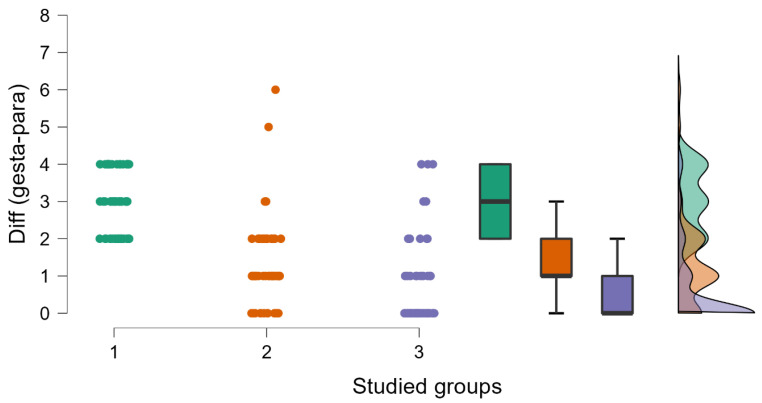
The differences between the number of pregnancies and miscarriages in all studied groups, using raincloud plots. A significant decrease was observed (*p* < 0.001, *p* value obtained from Kruskal–Wallis test; groups 1 and 2 contain 51 patients each, group 3 contains 76 patients).

In Table 1, the impact of age, the body mass index (BMI), the gestational period (GP), and the newborns’ weight are presented. It may be observed that the differences appear in the GP and the weight of the newborns (see Figure 1 and Figure 2). If we study the number of pregnancies and miscarriages, we observe that higher values are detected in the case of inherited thrombophilia (see Figure 3). Regarding the environmental setting, 79.78% of our patients were from an urban environment having a median age higher than those living in rural areas (32 years old vs. 28 years old), an outcome that is expected for a Western type of society [16]. A 2019 study on 818 pregnant women [17] determined that the mean age of the enrolled pregnant woman diagnosed with thrombophilia who used LWMH during pregnancy was 33.9 years old, with an SD ± 4.9 years. Similar to these findings, our patients have approximately the same age range (group 1—33 years old, with an IQR ± 5 years, group 2—30 years old, with an IQR ± 7 years, and group 3—30.5 years old, with an IQR ± 7 years). The risk of maternal and fetal difficulties during pregnancy, such as stillbirth, small-for-gestational-age births, preterm birth, preeclampsia, and maternal death, increases in women as age enhances, presenting as hazard gain as age increases [18]. Smoking is one of the most important risk factors for worse pregnancy outcomes [19], and it affected all three groups of our study by aggravating some biological changes such as an increase in D-dimers levels during the last trimester of pregnancy and a decline in antithrombin levels; we also obtained that pregnant smokers needed higher doses of anticoagulant, approximatively over 20%. We tested the D-dimers evolution during pregnancy for all the studied groups and we registered a significant increase in our values in all scenarios, regardless of the type of thrombophilia that our patients had.

According to the objectives of the study, to highlight the particularities of thrombophilia in the western part of Romania, the characteristic profile of the patient with thrombophilia regarding the anthropometric parameters provides an average age of the patients of 32.8 years in the first group, 31 years in the second group, and 31.3 years in group 3, an average BMI value of 22.9 in the first group, 22.2 in the second group, and 22.9 in the third group, and an average gestational period of 37.3 weeks in the first group, 35.8 weeks in the second group, and 35.4 weeks in the third group. In terms of the average weight of the newborn, we observe a value of 2842.2 g in the first group, 2724.5 g in the second group, and 2634.5 g in the third group. To properly provide therapies for preterm labor and premature infants, accurate gestational dates during pregnancy are necessary; we also investigated this aspect, and we obtained that the gestational period (GP) and the newborns’ weights show discrepancies.

To monitor the treatment response during pregnancy, we ran some specific medical tests such as D-dimers (ng/mL) and the levels of antiXa (anti Xa levels UI/mL) in all three pregnancy trimesters; the results are presented in Table 2. To establish the acquired form of thrombophilia, we ran the tests presented in Table 3. 

The analysis of the genetic markers is presented in Table 4. In terms of the genetic markers involved, the highest frequency is represented by the MTFHR gene mutation C677T and A1298C, followed by the PAI-1 4G/5G gene mutation and the factor XIII gene mutation. In our research, the MTHFR_C677T mutation was homozygous in 11.76% of the cases from group 1 and heterozygous in 52.95% of the cases from the same group. The MTHFR_A1298C mutation was heterozygous in 35.29% of group 1 and 46.05% of group 3. We found the homozygote form of PAI-1 4G/5G in 31.37% of our patients diagnosed with hereditary thrombophilia, and in 22.37% diagnosed with the mixed type. Likewise, the women from our study diagnosed with mixed thrombophilia tested positive in 3.95% of the cases for the homozygote form of ACE_insertion_deletion mutation (angiotensin converting enzyme gene)**,** which was demonstrated to increase miscarriage risk in European women [20,21]. We obtained that 10.53% of the women diagnosed with mixed thrombophilia in our analysis tested positive for the heterozygous form of this mutation. However, early pregnancy loss is linked to homozygosity for the FXIII 34Leu polymorphism [22], which was the case in 13.72% of our patients (diagnosed with inherited thrombophilia, the factor XIII G1002T mutation (Val34Leu) was in the homozygote form).

**Figure 4:**
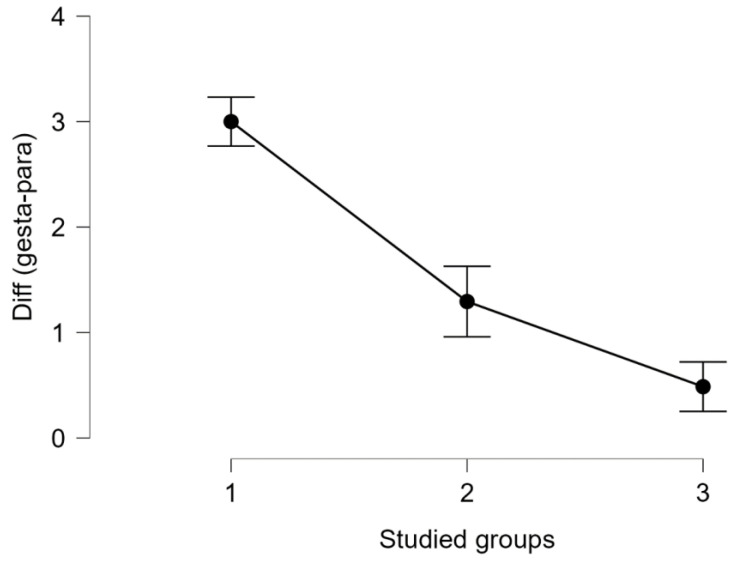
The miscarriage risk, compared between our groups. A significant decrease can be observed within the studied groups, and the lowest rate was reported in group 3 (*p* < 0.001, *p* value obtained from Kruskal–Wallis’s test; groups 1 and 2 contain 51 patients each, group 3 contains 76 patients).

**Figure 5:**
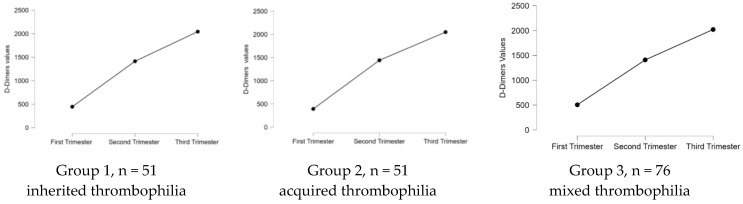
The D-dimers evolution during pregnancy, based on the type of thrombophilia (groups 1, 2, and 3—*p* < 0.001, *p* value obtained from the Friedman test).

The most frequent form of thrombophilia found in our study was the mixed one (42.7%). None of the patients was diagnosed with APS. Regarding the genetic factors involved in thrombophilia, it is important to start monitoring an early pregnancy of a woman diagnosed with this pathology to know when to start the proper treatment. Nevertheless, a 2002 study presented that mothers who have the factor V G1691A or factor II A (20210) mutation are far more likely to give birth to babies who are underweight at birth [23]. All women with a known personal history of preeclampsia, recurrent miscarriages, fetal growth restriction, first-trimester abortion, mid-trimester abortion, placental abruption, or intrauterine mortality should undergo a clinical and paraclinical examination for thrombophilia [7,24,25]. We applied the Mann–Whitney U-test to see if there were statistically significant differences in the age of our patients based on their environment, and we obtained significant differences $\left(p<0.001\right)$ in all three groups (see Table 5, Appendix A); the age of the patients who were from an urban environment is significantly higher compared with the patients from a rural area. This can be explained by the fact that patients from urban areas tend to form families later than those from rural areas. To quantify the impact of thrombophilia type in miscarriages, we applied a Kruskal–Wallis test, and we obtained significant differences $\left(p<0.001\right).$ The highest chance of a miscarriage was registered in the case of inherited thrombophilia (see Figure 4). We applied the Friedman test to see the D-dimers evolution in the pregnancy trimesters for all the studied groups. We obtained in all scenarios a significant increase $\left(p<0.001\right)$ in our values, regardless of the type of thrombophilia that our patients had. All the results are plotted in Figure 5.

## 4. Discussion

In our research, our results show that women diagnosed with thrombophilia are more likely to suffer pregnancy losses. Studying thrombophilic conditions will help us develop preventative strategies. This could be a new area of study for Romania’s western region addressing women who have experienced previous miscarriages and could undergo a national screening protocol. A wide variety of coagulation and genetic tests are required to be performed, and their interpretations necessitate clinical knowledge. Therefore, women ought to, at the very least, undergo genetic testing.

Analysis of the miscarriage risk shows that women who had a history of losses before the current pregnancy were more likely to develop recurrent pregnancy loss (RPL) in the absence of appropriate treatment, confirming similar observations of other groups [26].

A meta-analysis of 81 case–control studies conducted in 2021 summarized that the risk of RPL may be greatly increased by the FVL mutation [27]; if the death was a stillbirth, FVL and fetal loss were significantly linked [28]. We found the heterozygote form of this mutation in 9.2% of our patients diagnosed with hereditary thrombophilia, and in 11.84% diagnosed with the mixed type.

Considered to be a crucial regulator of the fibrinolytic system is PAI-1. Therefore, any deviation from normal in this gene may impact hemostasis. Increased fibrin formation is linked to the PA1-1 4G/5G and may disrupt placenta circulation and implantation, potentially leading to pregnancy loss [20]. Pregnancy complications tested positive for the prothrombin G20210A mutation in 57.9% of patients [29] with an additional increased risk of early pregnancy loss and preterm placental abruption linked to the heterozygous GA variation [29,30].

According to research carried out in 2018 [22], women who experienced several miscarriages had a considerable increase in factor XIII V34L mutations.

Hyperhomocysteinemia is primarily caused by MTHFR gene polymorphism [31]. Dai et al. hypothesized that excessive homocysteine levels and the MTHFR 677CT and 1298AC genotypes together enhanced the aberrant lipid metabolism in RPL patients [31], so this may be a further direction of research for the western part of Romania regarding women with a history of miscarriages. We prescribed thrombolytics, namely Aspirin, to women with antithrombin, protein C, or protein S deficiency, following recommendations of a certain study in which aspirin enhances implantation and placentation and has vasodilatory effects through boosting prostacyclin production, according to multiple earlier studies [32]. Aspirin may help endothelial dysfunction and appears to have a direct impact on platelets [33].

Smoking is a risk factor manifested in all three groups by the aggravation of some characteristic biological changes, such as the increase in D-dimers values in the last trimester of pregnancy and the decrease in antithrombin values. We applied the Mann–Whitney U-test to the thrombophilia-specific medical tests to see if smoking habit can influence the parameter of thrombophilia-specific medical tests. We evaluated in groups a part of it as well as the whole sample and we obtained significant results $\left(p<0.05\right)$ in the case of LMWH dose (ml) in all three groups (group 1, p=0.048; group 2, p=0.046; group 3, p=0.038); the values of smoker patients are significantly higher. We have significant results $\left(p<0.05\right)$ in the case of D-dimers ng/mL in the third trimester of pregnancy in all three groups (group 1, p=0.019; group 2, p=0.023; group 3, p=0.013); the smoker patients had higher values. Finally, significant results $\left(p<0.05\right)$ were obtained in the case of antithrombin values in all our groups (group 1, p=0.009; group 2, p=0.003; group 3, p=0.007); the smoker patients in this case had significantly lower values. When we analysed the antithrombin values within our groups, we obtained significant differences $\left(p=0.045<0.05\right)$, the lowest values being registered in the second group—acquired thrombophilia; in groups 1 and 3 we observed almost the same mean values.

Combining preventive dosages of LMWH and aspirin starting before the second trimester of pregnancy has been shown to minimize the incidence of miscarriage in women with genetic thrombophilia [34,35]. LMWH dose adjustments during pregnancy by antifactor Xa activity levels were typical in this retrospective observational cohort analysis of gravidas maintained on LMWH for prophylaxis or treatment of VTE. More frequent monitoring of antifactor Xa levels is recommended in pregnant patients for which a specified target of antifactor Xa is aimed for [36,37].

Various studies [5,38,39] have suggested that certain thrombophilic variants are linked to miscarriages. All of these matched the findings of our study.

## 5. Conclusions

From the point of view of the thrombophilia profile performed in the western part of Romania, the mixed type of thrombophilia predominates. The particularities of pregnant women diagnosed with thrombophilia are higher age, living in an urban environment, normal BMI, approximately 36 weeks of gestational period, and having at least one miscarriage. Regarding the most frequent thrombophilic genetic markers, we obtained the MTFHR gene mutation C677T and A1298C, followed by the PAI-1 4G/5G gene mutation. Another important aspect was the impact of the smoking habit in thrombophilic pregnant women: smoking represents an aggravating factor in the evolution of this pathology, manifested through the increase of D-dimers and the decrease in antithrombin values, simultaneously with the increase in therapeutic need.

## Figures and Tables

**Table 1:** Descriptive analysis upon anthropometric characteristics (IQR—interquartile range).

Variables	Age (Years)			BMI (G (kg)/h^2^ (m^2^)			GP (Weeks)			Newborn Weight (g)		
	1	2	3	1	2	3	1	2	3	1	2	3
Valid	51	51	76	51	51	76	51	51	76	51	51	76
Missing	0	0	0	0	0	0	0	0	0	0	0	0
Mean	32.8	31	31.3	22.9	22.2	22.9	37.3	35.8	35.4	2842.2	2724.5	2634.5
Median	33	30	30.5	20.8	19.8	20.8	38	37	36	3100	2700	2780
Minimum	21	23	23	15.9	17.2	15.9	30	30	30	1240	1300	1240
Maximum	45	49	49	34.9	34.9	34.9	40	40	42	3770	3740	3880
IQR	5	7	7	6.8	3.8	5.8	1	6	6	660	1000	935

**Table 2:** The quarterly evolution of the parameters studied for therapeutical orientation (95% confidence interval (median ± 1.5 IQR); IQR—interquartile range).

Medical Tests	95% Confidence Interval		
	Group 1 (n = 51)	Group 2 (n = 51)	Group 3 (n = 76)
D-dimers ng/mL—Trim1	(102.87; 790.87)	(116.3; 672.3)	(121.82; 889.82)
D-dimers ng/mL—Trim2	(1108.3; 1719.7)	(1106.8; 1778.3)	(1105.5; 1715.3)
D-dimers ng/mL—Trim3	(1688.8; 2400.5)	(1690.5; 2409.9)	(1637.1; 2408.2)
anti Xa levels UI/mL—Trim1	(0.24; 0.52)	(0.13; 0.77)	(0.10; 0.79)
anti Xa levels UI/mL—Trim2	(0.26; 0.44)	(0.22; 0.44)	(0.21; 0.43)
anti Xa levels UI/mL—Trim3	(0.35; 0.48)	(0.34; 0.47)	(0.29; 0.47)

**Table 3:** The analysis of the parameters involved in tests performed to establish existence of the acquired form of thrombophilia (95% confidence interval (median ± 1.5 IQR); IQR—interquartile range).

Medical Tests	95% Confidence Interval		
	Group 1 (n = 51)	Group 2 (n = 51)	Group 3 (n = 76)
Lupus Anticoagulant (ratio)	(1.03; 36.35)	(0.97; 33.09)	(0.05; 44.77)
Anti Cardiolipin antibodies IgG (U/mL)	(0.25; 2.49)	(0.16; 2.2)	(0.14; 1.9)
Anti Cardiolipin antibodies IgM (U/mL)	(0.02; 1.5)	(0.05; 1.21)	(0.01; 1.29)
Protein C (%)	(6.92; 131.48)	(1.28; 120.88)	(20.6; 132.2)
Protein S (%)	(4.3; 106.3)	(0.7; 98.3)	(3.9; 115.5)
Antithrombin (%)	(15.77; 122.57)	(3.99; 104.15)	(24.23; 123.83)
Homocysteine (micromole/L)	(0.16; 8.56)	(0.13; 9.73)	(0.1; 10.5)

**Table 4:** Analysis of the genetic markers of thrombophilia in the three studied groups.

Mutation	Group 1 (n = 51)		Group 2 (n = 51)		Group 3 (n = 76)	
	Present	Absent	Present	Absent	Present	Absent
MTHFR_C677T	11.76% homozygote 52.95% heterozygote	35.29%	0%	100%	13.16% homozygote 56.58% heterozygote	30.26%
MTHFR_A1298C	7.84% homozygote 35.29% heterozygote	56.86%	0%	100%	6.58% homozygote 46.05% heterozygote	47.37%
Factor V Leiden	9.2% heterozygote	90.2%	0%	100%	11.84% heterozygote	88.16%
Prothrombin gene G20210A (Factor II)	1.96% heterozygote	98.04%	0%	100%	10.53% heterozygote	89.47%
Glycoprotein IIb/IIIa T1565C	13.72% heterozygote	86.28%	0%	100%	10.53% heterozygote	89.47%
PAI_1_4G/4G	0%	100%	0%	100%	9.21% homozygote	90.79%
PAI_1_4G_5G	31.37% homozygote 39.22% heterozygote	29.41%	0%	100%	22.37%homozygote 35.53% heterozygote	42.11%
PAI_1_5G_5G	0%	100%	0%	100%	6.58% homozygote 1.32% heterozygote	92.11%
FactorV_R2	1.96% heterozygote	98.04%	0%	100%	17.11% heterozygote	82.89%
B_Fibrinogen G455A	7.84% heterozygote	92.16%	0%	100%	3.95% homozygote 7.89% heterozygote	88.16%
ACE_insertion_deletion (angiotensin converting enzyme gene)	0%	100%	0%	100%	3.95% homozygote 10.53% heterozygote	85.53%
AGT mutationM235T (the angiotensinogen gene)	0%	100%	0%	100%	2.63% homozygote 11.84% heterozygote	85.53%
FactorV 4070_AgtG	0%	100%	0%	100%	2.63% homozygote	97.37%
factor XIII G1002T mutation (Val34Leu)	13.72% homozygote 23.53% heterozygote	62.75%	0%	100%	2.63% homozygote 40.79% heterozygote	56.58%
ATR-1 Mutation A1166C (serine/threonine kinase gene)	0%	100%	0%	100%	1.32% homozygote 7.89% heterozygote	90.79%
CBS 844ins68 (the Cystathionine Beta-Synthase gene)	0%	100%	0%	100%	2.63% homozygote	97.37%

**Table 5:** Comparative analysis of thrombophilia-specific parameters (groups 1 and 2 contain 51 patients each, group 3 contains 76 patients).

Variables in Study	Type of Test	Obtained *p* Value	Conclusion
Differences in age based on the patient’s environment	Mann–Whitney U-test	p<0.001—group 1	The age is higher for the patients who are living in urban areas
		p<0.001—group 2	
		p<0.001—group 3	
LMWH dose (mL) for smokers and nonsmokers	Mann–Whitney U-test	p=0.048—group 1	Smokers have higher values for LMWH doses in all groups
		p=0.046 —group 2	
		p=0.038—group 3	
D-dimers values in the third trimester of pregnancy for smokers and nonsmokers	Mann–Whitney U-test	p=0.019—group 1	Smokers have higher values of D-dimers in all groups
		p=0.023—group 2	
		p=0.013—group 3	
Antithrombin values in the third trimester of pregnancy for smokers and nonsmokers	Mann–Whitney U-test	p=0.009—group 1	Smokers have lower values of antithrombin in all groups
		p=0.003 —group 2	
		p=0.007—group 3	
Thrombophilia type—the impact in miscarriages	Kruskal–Wallis test	p<0.001	The highest chance for a miscarriage is in the case of inherited thrombophilia
The antithrombin values within the three groups	Kruskal–Wallis test	p=0.045	The lowest values are in the second group—acquired thrombophilia
The D-dimers evolution during the pregnancy trimesters for all three groups	Friedman test	p<0.001—group 1	The D-dimers values are increasing significantly in all three groups
		p<0.001—group 2	
		p<0.001—group 3	

## Data Availability

The use of the database was possible with the agreement of the Bioethics Commission.

## Appendix A

A raincloud plot was used in order to obtain a complete picture of data dynamics (when we had to analyze the difference between two samples (e.g., environment, smoker), the *p* value was obtained from the Mann–Whitney U-test; when we had to analyze the differences between the studied groups, we applied the Kruskal–Wallis test).

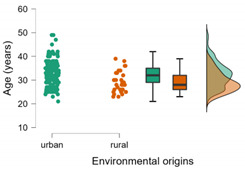
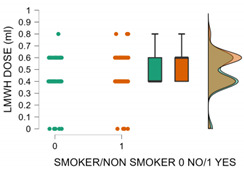
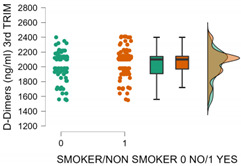

		
Differences in age based on the patient’s environment, a significant decrease was observed in the age of patients coming from a rural environment (*p* < 0.01).	LMWH dose (mL) for smokers and nonsmokers—a significantly higher value of LMWH was registered in smoking patients (*p* = 0.048).	D-dimers values in the third trimester of pregnancy for smokers and nonsmokers; a significantly higher value was registered in smoking patients (*p* = 0.013).

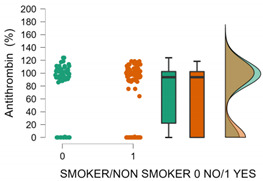
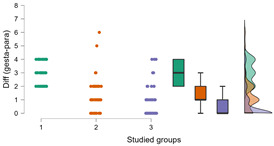
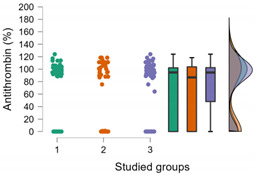
		
Antithrombin values in the third trimester of pregnancy (smokers and nonsmokers). A significant decrease was observed in smoking patients (*p* = 0.007).	Thrombophilia type—the impact in miscarriages—a significant decrease was observed in the third group (*p* < 0.001).	The antithrombin values within the three groups. A significantly lower antithrombin value was registered in the second group (*p* = 0.045).
